# Potential Role of Natural Plant Medicine *Cyclocarya paliurus* in the Treatment of Type 2 Diabetes Mellitus

**DOI:** 10.1155/2021/1655336

**Published:** 2021-12-27

**Authors:** Han Wang, Cheng Tang, Zezheng Gao, Yishan Huang, Boxun Zhang, Jiahua Wei, Linhua Zhao, Xiaolin Tong

**Affiliations:** ^1^Guang'anmen Hospital, China Academy of Chinese Medical Sciences, China; ^2^Changchun University of Chinese Medicine, China

## Abstract

Type 2 diabetes mellitus (T2DM) is a common chronic metabolic disease that has become increasingly prevalent worldwide. It poses a serious threat to human health and places a considerable burden on global social medical work. To meet the increasing demand for T2DM treatment, research on hypoglycemic drugs is rapidly developing. *Cyclocarya paliurus* (Batal.) Iljinskaja is a medicinal plant that grows in China. The leaves of *C. paliurus* contain polysaccharides, triterpenoids, and other chemical components, which have numerous health benefits. Therefore, the use of this plant has attracted extensive attention in the medical community. Over the past few decades, contemporary pharmacological studies on *C. paliurus* extracts have revealed that it has abundant biological activities. Multiple in vitro and in vivo experiments have shown that *C. paliurus* extracts are safe and can play a therapeutic role in T2DM through anti-inflammatory and antioxidation activities, and intestinal flora regulation. Its efficacy is closely related to many factors, such as extraction, separation, purification, and modification. Based on summarizing the existing extraction methods, this article further reviews the potential mechanism of *C. paliurus* extracts in T2DM treatment, and we aimed to provide a reference for future research on natural plant medicine for the prevention and treatment of T2DM and its related complications.

## 1. Introduction

The prevalence of diabetes has been increasing globally. According to the International Diabetes Federation, there were 463 million diabetic patients in 2019, and this number is expected to reach 578.4 million by 2030. Among the diabetic patients, about 90-95% is T2DM [1]. The shortage of medical resources, the difference in levels of technology, and the gap between the rich and the poor have brought challenges to disease control. Existing research on the mechanism to fully clarify the etiology of the disease is insufficient. Available evidence shows that insulin resistance (IR) and *β*-cell dysfunction are two major pathological characteristics of the disease [2]. As a result, patients often have elevated blood glucose levels due to insufficient insulin secretion or utilization. If blood glucose cannot be controlled in a timely and effective manner, it will lead to a series of life-threatening complications, such as renal failure, heart disease, amputation, and blindness [3].

In the past decades, the potential of traditional Chinese herbal medicines in diabetes treatment has gradually been recognized and accepted by the medical community. In contemporary pharmacological research, an increasing number of natural plants have been explored and applied for their medicinal value. The Juglandaceae plant *C. paliurus* is also known as a sweet tea tree because of its unique sweetness. This plant is primarily distributed in the subtropical plateau of southern China. According to the literature, the leaves, seeds, and bark of *C. paliurus* can be used medicinally. At present, *C. paliurus* leaves are generally accepted as health care products or medicines by local and medical communities [4], and dozens of compounds have been extracted from the plant. Previous research has confirmed that the plant has numerous biological activities, such as hypoglycemic, antihypertensive, lipid-lowering, anticancer, antioxidant, antibacterial, hepatoprotective, and colon health [5–8]. Through further investigation of the extracts, researchers proved that the biological activity of plants was affected by geographical location, molecular content, and molecular structure [9, 10]. Furthermore, these compounds were affected by the method of extraction.

As a natural plant medicine, *C. paliurus* has a clear and sweet taste and can be easily consumed by the majority of patients. Moreover, this plant is relatively safe. There are no reports of hepatorenal toxicity or obvious side effects in cases where this plant is used in large doses [11–14]. The medicinal value of *C. paliurus* presents considerable potential in various diseases. Therefore, *C. paliurus* may also have great potential in the field of medicine.

Although there is substantial evidence that *C. paliurus* can alleviate diabetes in several ways, no study has systematically summarized the role of this natural plant in diabetes treatment. This paper reviews the literature on the extraction methods and therapeutic mechanisms of *C. paliurus*, and we aimed to provide new ideas and directions for the application of *C. paliurus* in T2DM and its related complications in future research.

## 2. Extraction of Effective Components from *C. paliurus*

To obtain the effective components of *C. paliurus*, various extraction methods should be adopted. These methods mainly include crude extraction, optimized extraction, separation, and purification. In addition, it has been confirmed that the structure of compounds can be further ameliorated by modification, and biological activities can be improved [15].

### 2.1. Extraction and Optimized Extraction

Existing studies have supported the use of *C. paliurus* leaves to explore its medicinal value. To date, the extraction of *C. paliurus* predominantly includes water extraction, ethanol extraction, ultrasonic-assisted extraction, and the hypoglycemic mechanism of polysaccharide microwave-assisted extraction. Among them, hot water and ethanol are the main methods used for preliminary crude extraction to obtain the effective components [16]. Nevertheless, there are certain limitations to these traditional approaches, such as time consumption, high-temperature requirements, and low extraction efficiencies. To optimize the extraction outcome, researchers have used ultrasonic and microwave-assisted methods to obtain more effective extracts. It has been confirmed that the *C. paliurus* polysaccharides after ultrasonic treatment have better scavenging activity against 1,1-diphenyl-2-picrylhydrazyl (DPPH) and hydroxyl radicals, indicating that ultrasonic treatment can promote the antioxidant activity of *C. paliurus* polysaccharides [17]. Another study reported that polysaccharides extracted by ultrasonic-assisted extraction have the advantages of being highly efficient, less solvent, and less time consuming and has the function of scavenging free radicals and inhibiting lipid peroxidation to a certain extent [8]. Xie et al. found that the microwave-assisted extraction method also has clear advantages in the extraction of *C. paliurus* polysaccharides, featuring high extraction efficiency and reduced time consumption [18]. The compounds obtained were also different under the influence of different extraction methods. A study showed that water extract is rich in polysaccharides, while ethanol extract is rich in triterpenoids and flavonoids [9].

### 2.2. Separation and Purification

Crude extracts generally contain a variety of impurities, such as low-molecular-weight compounds and proteins; therefore, they are separated and purified after preliminary extraction. The ethanol precipitation method has the advantage of simple steps; however, this method is not conducive to purification and consumes a large amount of organic solvents [19]. Ultrafiltration is a common separation method that has the advantages of high efficiency, low cost, and environmental friendliness, and after crude extraction and separation, certain low-molecular-weight compounds can be removed; however, extracts still require further purification by ultrapure water dialysis [20], Sevage method [21], decolorization, and chromatography [22].

### 2.3. Modification

Elevated levels of chronic oxidative stress markers appear in the early stages of IR or T2DM [23]. In addition, a variety of inflammatory chemokines can be used as predictive markers of T2DM [24]. Modern studies have confirmed that although traditional Chinese medicine (TCM) has anti-inflammatory and antioxidant effects, its pharmacological effects can be significantly enhanced through structural modification [25, 26]. The modification of compounds is considered to improve their utilization value. At present, the modification of *C. paliurus* mainly includes acetylation, sulfation, phosphorylation, and carboxymethylation. Liu et al. demonstrated that acetylated *C. paliurus* polysaccharides have better immunomodulatory activity [27]. Sulfated polysaccharides exhibit outstanding anti-inflammatory and antioxidative stress properties. Han et al. elucidated that sulfated *C. paliurus* polysaccharides enhanced the immunomodulatory activity of dendritic cells through the TLR2/4-mitogen-activated protein kinase (MAPK)/nuclear factor kappa-B (NF-*κ*B) signaling pathway. Moreover, after sulfation, phosphorylation, and carboxymethylation modification, the antioxidant capacity of polysaccharides was also improved [28, 29]. Appropriate modification plays a vital role in the improvement of biological activities; however, according to the existing literature search, the modification of *C. paliurus* extract mainly focused on polysaccharides. Therefore, it is necessary to explore more effective modification methods to better exert their biological activities.

Bioactive compounds with medicinal value have been successfully obtained using various extraction methods. According to existing research, common compounds primarily include polysaccharides, triterpenoids, flavonoids, phenolic acids, and saponins. Meanwhile, a small number of carbohydrates, proteins, mineral elements, sterols, amino acids, organic acids, and unsaturated fatty acids are present in this plant. [4, 30–32]. These active substances are the material basis of drug efficacy.

## 3. The Therapeutic Effect of *C. paliurus* on T2DM

Following centuries of application, *C. paliurus* has been recognized to exhibit the benefits of regulating glucose and lipid metabolism. In vitro and in vivo studies have shown that water and ethanol extracts can reduce blood glucose and lipid levels in T2DM patients. Among these compounds, *C. paliurus* flavonoids have a potential hypoglycemic effect, triterpenoids have a more obvious lipid-lowering effect [9], and quercetin and kaempferol are the main factors that inhibit *α*-glycosidase activity. This inhibition ability is even stronger than that of acarbose [33]. In addition, they can reduce serum total cholesterol (TC) and triglycerides (TG), ameliorate liver fat levels, inhibit pancreatic lipase activity, reduce body weight, and improve liver steatosis [34].

## 4. Summary on Potential Mechanism of *C. paliurus* on T2DM (Figure 1)

### 4.1. Improvement of IR

The majority of T2DM patients experience progression from IR to impaired islet *β*-cell function [35]. Therefore, alleviating IR and protecting islet *β*-cell function are key points of T2DM treatment. Lipid metabolism disorders and chronic inflammation caused by obesity are thought to be closely related to the occurrence of IR [36]. In addition, mitochondrial dysfunction, gut microbiota dysbiosis, and remodeling of the adipose extracellular matrix also play a bridging role between obesity and IR [37]. Xu et al. found that *C. paliurus* aqueous extract can inhibit energy intake in animal experiments, which may be due to hypothalamic insulin signaling pathway regulation and controlling proopiomelanocortin (POMC) and neuropeptide Y expression in order to inhibit excessive food intake [11]. In T2DM, the levels of hepatic TG in overweight and obese patients are directly related to the severity of IR in the liver and skeletal muscles [38, 39]. Recently, the effects of *C. paliurus* extracts on IR have attracted increasing attention. It has been shown that *C. paliurus* extracts can reduce IR and regulate the activity of key enzymes such as CCAAT/enhancer binding protein alpha and peroxisome proliferator-activated receptor *γ* in lipid metabolism [13]. In addition, in high-fat diet- (HFD-) induced mice and palmitic acid-induced HepG2 cells, it was found that *C. paliurus* triterpenoids (CPT) upregulated the phosphoinositide 3-kinase (PI3K)/protein kinase B (Akt)/glycogen synthase-3*β* (GSK3*β*) signaling pathway to alleviate IR [34]. The hepatolipid-lowering activity of *C. paliurus* extract is also reflected in mammalian target of rapamycin (mTOR)/70 kDa ribosomal protein S6 kinase (p70S6K) pathway regulation, lipophagy activation, and promotion of lipid decomposition in HepG2 cells [40]. In addition, CPT acts on the ng to improve insulin sensitivity of adipocytes and increase glucose intake, which may have potential as insulin sensitizers [41, 42]. Extensive research shows that, in T2DM, low chronic inflammation and IR are the two interdependent key processes; inflammation can interfere with insulin signaling; therefore, IR is closely related [43]. Persistent hyperglycemia can affect the inflammatory mechanism of the liver, leading to lipid accumulation and aggravated IR [44]. Jiang et al. found that *C. paliurus* extracts regulate adipokine expression and improve IR by inhibiting inflammation in mouse models [45]. These results provide support for the role of *C. paliurus* in improving insulin sensitivity and alleviating IR. This plant appears to be effective for T2DM treatment due to its effect on weight loss and IR alleviation.

### 4.2. Anti-Inflammatory and Antioxidative Stress

Substantial evidence has shown that oxidative stress and an increase in cytokines are significantly correlated with the occurrence and development of T2DM. Numerous cytokines, including interleukin-6 (IL-6), interleukin-1*β* (IL-1*β*), high-sensitivity C-reactive protein, and tumor necrosis factor-*α* (TNF-*α*), can act as central mediators of the inflammatory response and have been confirmed to be positively correlated with the risk of T2DM [46, 47]. Therefore, active intervention against proinflammatory cytokines is considered beneficial for T2DM treatment. Certain studies have shown that the hypoglycemic effect of triterpenoids may be mediated by the activation of adenosine 5′-monophosphate- (AMP-) activated protein kinase (AMPK), thereby inhibiting adipose tissue inflammation. Zhu et al. found that the chloroform extract of *C. paliurus* and its two triterpenoids can reduce inhibitor kappa kinase *β* (IKK-*β*) phosphorylation induced by inflammatory injury, and its anti-inflammatory effect may be related to AMPK activation [42]. The compound polysaccharide and dammarane triterpene saponin from *C. paliurus* has also been proven to be conducive to proinflammatory cytokine inhibition and inflammatory pathway regulation, and research has demonstrated that the expression and release of nitrate oxide, TNF-*α*, and prostaglandin E2 (PGE2) were significantly inhibited in lipopolysaccharide- (LPS-) mediated inflammatory stimulation of RAW 264.7 [48–50]. Jiang et al. confirmed in vivo and in vitro that *C. paliurus* triterpenoids improve diabetes-induced liver inflammation through the Rho/Rho-associated coiled-coil-containing protein kinase (ROCK)/NF-*κ*B signaling pathway, and the expression of Rho kinase and NF-*κ*B in the liver was significantly reduced [51]. Transcriptome analysis showed that *C. paliurus* aqueous extract alleviated inflammation by inhibiting cytochrome P450 and enhancing fatty acid metabolism in diabetic rat livers, as evidenced by decreased TNF-*α* and IL-6 levels [52]. These results suggest that *C. paliurus* extracts might be beneficial for T2DM treatment through the regulation of inflammation.

It is widely acknowledged that oxidative stress is another major hallmark of T2DM. The most common markers of oxidative stress are malondialdehyde (MDA), superoxide dismutase (SOD), and glutathione (GSH). To a certain extent, the increase in reactive oxygen species (ROS) production can cause or aggravate diabetes by damaging *β*-cells, reducing insulin secretion, affecting glucose transport pathways, and negatively interfering with the balance of oxidant and antioxidant levels [53, 54]. Several clinical studies have shown that oxidative stress is directly related to IR, which can induce pancreatic *β*-cell apoptosis and cause abnormal glucose and lipid metabolism. Currently, exogenous antioxidants have gradually attracted attention for inhibiting apoptosis, enhancing autophagy, and improving oxidative stress [53]. Multiple studies have confirmed that the main polysaccharide compounds in *C. paliurus* have antioxidant activities. After using RNA-seq technology to study the polysaccharide synthesis pathway at different stages of leaf development and to detect the polysaccharide content and antioxidant activity, Liu et al. found that the hydroxyl scavenging activity was the highest and the antioxidant capacity was the strongest in the first stage [55]. The health beverage composed of *C. paliurus* polysaccharides and *Momordica* saponin has been proven in the worm model to ameliorate oxidative damage by reducing the levels of ROS, MDA, and nonesterified fatty acids [56]. Lin et al. demonstrated that *C. paliurus* polysaccharides effectively enhanced the stress resistance of *Caenorhabditis elegans* through longevity-promoting factor 1 and heat shock transcription factor 1, which may include the scavenging of free radicals and the alleviation of oxidative damage [57]. Another study showed that *C. paliurus* polysaccharides play an important role in scavenging free radicals and have the function of autoxidation of 1,2,3-pyrogallol, which has a significant effect on inhibiting lipid peroxidation [8]. For carbon tetrachloride- (CCl4-) induced oxidative stress in the livers and kidneys of mice, *C. paliurus* polysaccharides can reduce the induction of recombinant cytochrome P450 2E1 (CYP2E1) expression in the liver by CCl4 and then have a protective effect on the liver, which is mainly manifested by the reduction of ROS and MDA levels and the recovery of SOD and GSH peroxidase (GSH-PX) activities in the liver and kidneys [58]. In addition to polysaccharides, triterpenoids have also been proven to improve oxidative stress in vitro, exerting an antioxidant effect in HepG2 hepatic steatosis cells induced by free fatty acid (FFA) [59]. Compared with other *C. paliurus* compounds, polysaccharides exhibit good antioxidant activity, which has attracted attention in the medical field [60]; however, there is still a lack of extensive and in-depth mechanistic research.

### 4.3. Regulation of Gut Microbiota

The intestinal tract contains a microbiota composed of a large number of microorganisms, which directly affects the health of the host. As shown with a previous in-depth study, the intestinal microbiota plays an increasingly prominent role in the pathogenesis of T2DM [61]. At present, it is believed that regulation of the gut microbiome has a positive impact on blood glucose homeostasis and the prognosis of T2DM [62]. Clinically, certain active components of TCM or active ingredients isolated from plants have been shown to have potential regulatory effects on the microbiota [63, 64]. In vivo experiments on *C. paliurus* showed that the main effects of its compounds represented by polysaccharides and flavonoids were reflected in increasing short-chain fatty acid (SCFA) content, enriching intestinal and fecal microbiota diversity, and regulating the relative balance of dominant bacterial phyla and genera in the intestinal tract [17, 20]. Other studies have shown that the intervention of *C. paliurus* compounds represented by polysaccharides and flavonoids can be beneficial for the species diversity (*α* diversity and *β* diversity) of intestinal microbiota [65]. In terms of species composition of the microbiota, *C. paliurus* administration changed the composition of intestinal microbiota in mice/rats at multiple levels, for example, reducing the ratio of Firmicutes/Bacteroides, increasing the relative abundance of *Clostridia*, *rumenUCG-005*, and reducing the relative abundance of *Bacilli*, *Coriobacteriia*, *Lactobacillales*, *Faecalibacterium*, and *Mitsuokella*; simultaneously, the relative abundance of *Ruminococcaceae* and *Veillonellaceae* decreased in the community structure [20, 66, 67]. *Enterococcus faecalis* is a beneficial intestinal bacteria [68], and an acidic environment (low pH) can damage the membrane of *E. faecalis* to some extent [69, 70]. Transcriptome analysis showed that *C. paliurus* flavonoids could enhance the acid resistance of *E. faecalis* by downregulating the major facilitator superfamily transporter gene and other pathways, alleviating the negative effects caused by low pH, thus showing a positive impact on the production of intestinal probiotics [71]. Furthermore, *C. paliurus* polysaccharides can also help activate G-protein-coupled receptors, promoting intestinal L cells to secrete intestinal hormones GLP-1 and peptide tyrosinetyrosine (PYY), thus contributing to T2DM treatment [72]. *C. paliurus*-related compounds actively regulate the intestinal microbiota of animal models, and its therapeutic value on intestinal microbiota has been gradually clarified; however, there is still a lack of clear verification of specific pathways and targets of action, and the mechanism requires further investigation.

### 4.4. Protection of Islet *β*-Cells

As another important factor in the development of disease, the quantity and quality of islet *β*-cells directly affect the progression of T2DM patients. Therefore, the protection of islet *β*-cell function can largely assist in T2DM treatment. Based on transcriptome and biochemical analysis, it was previously found that *C. paliurus* formula extract reduced proinflammatory cytokines and islet damage, inhibited *β*-cell apoptosis, ensured normal insulin secretion, and reduced blood glucose in rat models [73]. Certain studies have reported that *C. paliurus* and its related extracts can effectively reduce the expression of proapoptotic factors caspase-8 and caspase-9 in the pancreatic tissue of streptozotocin- (STZ-) induced mice or rats, reduce the ratio of Bax/Bcl-2, effectively avoid islet *β*-cells apoptosis, and alleviate pancreatic injury, which may be related to the regulation of *C. paliurus* on the MAPK and Akt pathways [66, 74].

### 4.5. Regulation of Lipid Metabolism

Dyslipidemia is a major risk factor for T2DM. T2DM patients are often accompanied by dyslipidemia, which aggravates the risk of macrovascular complications [75]. As the initiating, inducing, and aggravating factors of T2DM, lipid metabolism disorders play a crucial role in the course of the disease. Abnormal glucose and lipid metabolism caused by hyperlipidemia and hyperglycemia aggravate the progression of metabolic diseases. A cross-sectional study of 4807 Chinese adults showed that 67.1% of T2DM patients had dyslipidemia [76]. Statins are one of the most widely used lipid-lowering drugs, which can effectively reduce blood lipid levels and reduce the occurrence of cardiovascular events [77]. Nevertheless, a significant number of patients may develop intolerance after receiving statins [78].

Numerous studies have found that certain elements in plants, such as *C. paliurus* leaves and related extracts, have significant effects in dyslipidemia treatment. Research shows that *C. paliurus* polysaccharides play a certain role in lipid-lowering in HFD-induced hyperlipidemic rat models, which is mainly reflected in the downregulation of fatty acid synthase (FAS) and hydroxymethylglutaryl-coenzyme A reductase (HMG-CoA) and the upregulation of the expression of triglyceride lipase (ATGL) and peroxisome proliferator-activated receptor *α* (PPAR*α*) [79], while reducing the deoxyribonucleic acid (DNA) methylation level of leptin and microsomal triacylglycerol transfer protein (MTTP) and downregulating the mRNA content of leptin and MTTP to ameliorate lipid metabolism disorders in rats [80]. Moreover, *C. paliurus* polysaccharides can reduce the whole-genome DNA methylation level of mouse liver induced by high-fat emulsion by regulating AMPK, adipocytokines, fatty acid metabolism, and other signaling pathways and play a lipid-lowering role in mouse models [81].

Using high-fat *C. elegans* as an animal model, the results showed that the polysaccharide-enriched extract from *C. paliurus* reduced the size and number of lipid droplets in *C. elegans* and reduced the accumulation of lipids through the monounsaturated fatty acid (MUFA) biosynthesis pathway, and the mediator 15 (MDT-15)/selenium binding protein-1(SBP-1) and nuclear hormone receptor NHR-49 (NHR-49)/MDT-15 signaling pathways [82]. In hyperlipidemic mice, *C. paliurus* chloroform extract improved the activity and gene expression of cholesterol 7*α*-hydroxylase (CYP7A1), inhibited 3-hydroxy-3-methyl glutaryl coenzyme A (HMG CoA) reductase, promoted the transformation of cholesterol to bile acid, and exerted a lipid-lowering effect [83].

Recent studies have shown that *C. paliurus* extract can prevent intestinal absorption of dietary fat by inhibiting the secretion of apoB48, thus effectively preventing hyperlipidemia and obesity [12]. In addition to polysaccharides, triterpenoids also play an important role in improving lipid metabolism disorders and are expected to be effective in regulating T2DM complicated with dyslipidemia. Wu et al. found that the triterpene acid-enriched components and ethanol extract of *C. paliurus* inhibited the excessive production and secretion of apoB48 in hyperlipidemic rats through the TNF-*α*/p38 MAPK signaling pathway [84, 85]. Wu et al. demonstrated that the triterpenoids of *C. paliurus* can also significantly reduce the oversecretion of apoB48 in Caco-2 cells [86]. Based on this, we hypothesized that *C. paliurus* has a therapeutic effect on T2DM complicated with dyslipidemia. However, the molecular mechanism has not been well established and requires further exploration and summary.

## 5. Application of *C. paliurus* in the Complication of T2DM

Previous studies have shown that *C. paliurus* plays a significant role in reducing blood glucose, alleviating IR, and regulating lipid metabolism. In vivo and in vitro experiments also showed that the plant exerts good effects in the treatment of diabetes-related complications. Ample evidence indicates that approximately 40% of T2DM is associated with diabetic nephropathy (DN) to varying degrees, and DN has become one of the main causes of end-stage renal disease (ESRD). The presence of DN significantly increases the risk of cardiovascular disease-related mortality, which has placed a considerable burden on the social health system [87]. The main features of DN include increased urinary albumin excretion, decreased glomerular filtration rate, persistent hyperglycemia, and persistent renal function decline. In terms of DN treatment, the benefits of angiotensin-converting enzyme inhibitors (ACEIs) and angiotensin receptor blockers have been recognized clinically and widely used to alleviate DN. Bergamo Nephrology Diabetes Complication Trial research shows that ACEIs have a protective effect on the kidney and can effectively prevent the production of microalbuminuria [88]. A meta-analysis showed that, compared with single-use, a low-dose combination of the two drugs can compensate for their respective shortcomings; therefore, it is more prominent in reducing urinary total proteinuria and urinary albumin excretion rate [89]. However, certain researchers have opposing views, they affirm the effectiveness of the combination of these drugs in reducing urinary protein; however, they emphasize that the potential risk of hyperkalemia cannot be ignored. In addition to hyperkalemia, patients also have a significantly increased risk of decreased renal function or renal failure [90]. The advantages of TCM in treating DN have become increasingly prominent [91]. A population-based cohort study included 125490 patients with DN, and the results showed that the incidence and mortality of ESRD in DN patients actively treated with TCM were lower than those without TCM treatment [92]. Recently, *C. paliurus* has been reported to be a promising therapy for DN. Studies have demonstrated that the extract of *C. paliurus* can improve the extensive thickening of the glomerular capillary basement membrane in HFD-STZ-induced diabetic rats and also assist in reducing the levels of blood urea nitrogen, creatinine, and glycated serum protein, which is beneficial for early-stage DN treatment [52, 93]. Xia et al. reported that *C. paliurus* polysaccharides can reduce blood glucose, improve renal function, enhance antioxidant capacity, and downregulate the expression of advanced glycosylation end products and transforming growth factor-*β*1, thus playing a protective role in DN rats [94]. Furthermore, autophagy-induced oxidative stress, inflammation, and apoptosis aggravate renal injury [95], and the *C. paliurus* triterpenic acid fraction regulates autophagy through the AMPK-mTOR pathway and reduces high glucose-induced HK-2 cell apoptosis [96]. Meanwhile, *C. paliurus* aqueous extract can inhibit oxidative stress and aldose reductase activity and effectively improve renal function and urinary protein excretion in DN rats, alleviating renal damage in diabetic rats [14].

Increasing evidence suggests that diabetic patients are at a higher risk of heart disease and heart failure. Currently, diabetic cardiomyopathy (DCM) is generally recognized as a cardiac structural and functional disorder in diabetic patients, excluding hypertension, coronary artery disease, or severe valvular heart disease [97].

As one of the most common pathogenic factors of diabetes, inflammation has been confirmed to be associated with DCM. NF-*κ*B is an important nuclear transcription factor in cells and is involved in the regulation of inflammation and myocardial cell injury. Simultaneously, NF-*κ*B activation can lead to the release of pro-inflammatory cytokines such as TNF-*α*, IL-1*β*, and IL-6, resulting in myocardial injury, dilated cardiomyopathy, and other myocarditis-related conditions [98]. Furthermore, cardiomyocyte apoptosis may also be involved in the occurrence and progression of DCM [99]. To date, there is no specific medicine for targeted treatment of the disease. In clinical practice, individualized interventions are adopted based on controlling blood glucose levels. Following intragastric administration of *C. paliurus* ethanol extract to db/db mice, Wang et al. found that the levels of myocardial injury markers (cardiac troponin I and creatine kinase MB), oxidative stress markers (MDA and SOD), and proinflammatory cytokines (TNF-*α*, IL-1ß, and IL-6) were effectively alleviated. Further mechanistic exploration revealed that *C. paliurus* extract reduced the expression of NF-*κ*B and myocarditis by activating the PI3K/Akt signaling pathway, improving antiapoptotic Bcl-2, and reducing prapoptotic cle-caspase-3, cle-caspase-9, and Bax, to reduce myocardial inflammation and injury in mouse models. Therefore, it is speculated that it may effectively reduce pathological damage and fibrosis of the heart tissue in diabetic patients [100].

These results indicate that *C. paliurus* is a good target for the treatment of DN and DCM. However, the application of other diabetic complications requires further improvement, and the specific components of the corresponding bioactivities in the extract require further exploration.

Current research on the prevention of diabetes complications by *C. paliurus* extract is still in its initial stages. Xiao et al. found that after *C. paliurus* intervention, in addition to the effective improvement of biochemical indexes such as liver and kidney function, pathological changes such as glomerular basement membrane thickening, hepatic steatosis, and myocardial hypertrophy in model mice have also been effectively alleviated. *C. paliurus* has remarkable potential in the treatment of diabetes mellitus and various related complications, such as inflammation, oxidative stress modulators, and apoptosis inhibitors, which are worth exploring in depth.

## 6. Conclusion and Perspectives

As a natural plant, *C. paliurus* mainly grows in southern China and plays an important supporting role in numerous metabolic diseases, such as diabetes, obesity, and hypertension. The extracted plant components have been shown to have abundant biological activities in vivo and in vitro.  Table 1 summarizes the potential effects of major compounds and extracts from *C. paliurus* on T2DM and related complications.

This study focused on the application of *C. paliurus* in T2DM and its related complications, with particular focus on the acquisition of effective components and extracts. The extraction method of the active components directly affects biological activities. Researchers should select the extraction method according to the purpose, requirements, and conditions of the study and further optimize the extraction process. The development of more efficient separation and purification technologies will effectively prevent the loss of effective plant components and bring benefits for the next step of pharmacological value research. Through a review of the existing literature, it was found that *C. paliurus* has the characteristics of multiple targets and pathways in T2DM treatment. It was found that the compounds represented by polysaccharides and triterpenes and the extracts represented by water extraction and ethanol extraction had unique advantages.

For instance, *C. paliurus* polysaccharides have a significant therapeutic effect on T2DM and can effectively inhibit *β*-cell apoptosis, regulate intestinal flora, and exert anti-inflammatory and antioxidant effects. *C. paliurus* triterpenoids play a significant role in alleviating IR and regulating lipid metabolism and can be used as a promising effective component in T2DM complicated with lipid metabolism disorder or obesity. Moreover, existing research has shown that *C. paliurus* polysaccharides and triterpenoids have significant effects on the regulation of inflammation and oxidative stress. As for the regulation of gut microbiota as a way of antidiabetes, *C. paliurus* flavonoids and polysaccharides are more prominent. However, certain studies only proposed the bioactivities of the components obtained by different extraction methods and did not further detect the exact components, and further research on the main chemical components and mechanisms of biological activities may become a focus in the next stage.

Existing studies have shown that the natural product *C. paliurus* can have a good curative effect while being safe compared with the limitations of adverse reactions, drug resistance, toxicity, and side effects of commonly used modern drugs. At present, there are no reports of toxicity or liver injury. Surprisingly, many animal experiments have confirmed that *C. paliurus* extracts have a hypoglycemic effect similar to that of metformin. In addition, studies have shown that the inhibitory activity of *C. paliurus* extracts on glucosidase is even stronger than acarbose. The authors of this paper speculate that it may become the main component of drugs or functional foods to supplement the shortcomings of traditional therapies. Notably, all existing studies on *C. paliurus* are based on animals or cells and lack randomized controlled trials. Due to the differences among different species, although much evidence shows that *C. paliurus* has considerable potential in rodent research, its effectiveness in humans remains to be further established.

*C. paliurus* is widely used as a health drink, and its medicinal value requires further promotion and application. There are no reports on the dose-response relationship of *C. paliurus* as a therapeutic drug. Further exploration of *C. paliurus* is needed to fill these gaps, and the evaluation of *C. paliurus* as a means of intervention still requires further research.

## Figures and Tables

**Figure 1:**
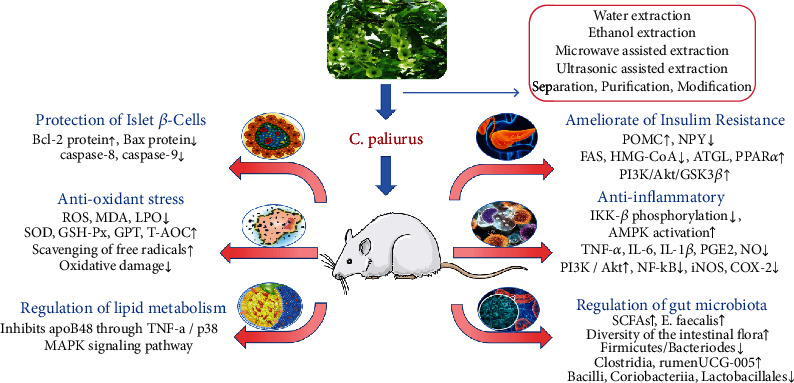


**Table 1:** Potential role of the major compounds and extracts of *C. paliurus* in T2DM and its complications.

Chemical compound/extract	Method of extraction	Model for the experiments	Effect	Potential mechanism	References
Polysaccharide	Water extraction	Male Wistar rats	Regulation of gut microbiota	UCG-005, SCFAs↑ IL-1*β*, IL-6, TNF-*α*↓ Leptin↓, adiponectin, GLP-1↑ Bcl-2↑, Bax↓	[66]
	Ethanol extraction	Sprague-Dawley rats	Regulation of gut microbiota	SCFA-producing bacteria↑ mRNA in GPR41, GPR43, GPR109a↑ SCFA-GLP1/PYY associated sensory mediators↑	[72]
	Ethanol extraction	Sprague-Dawley rats	Hypolipidemic	FAS, HMG-CoA↓, ATGL, PPAR↑	[79]
	Ethanol extraction	Sprague-Dawley rats	Hypolipidemic	DNA methylation of leptin and MTTP↓, mRNA contents of leptin and MTTP↓	[80]
	Hot water extraction	RAW264.7 cell	Anti-inflammatory	Accounted for synergistic effect on the release of NO and TNF-*α*, accounted for antagonistic effect on the release of IL-1*β* and PGE2.	[50]
	Hot water method assisted by ultrasonic	Female ICR mice	Hypolipidemic	The expression of ATGL and PPAR*α* gene in liver↓, high expression level of fatty acid synthesis gene induced by HFD↓, SOD, GSH-PX, GPT, T-AOC↑, LPO, MDA↓	[13]
	Water extraction	Kunming mice	Regulation of gut microbiota	SCFAs↑, diversity of the intestinal flora↑, specific metabolic functions of the gut microbiota↑	[20]
	Water extraction	C. elegans	Hypolipidemic	Size distribution of lipid droplets↓, number of lipid droplets↓, through MUFA biosynthetic pathways, MDT-15/sbp-1 and NHR-49/MDT-15 signaling pathway	[82]
	Ethanol extraction	Kunming mice	Antioxidant	Induction of CYP2E1 expression in liver by CCl4↓, ROS↓, SOD, GSH-Px↑, MDA↓	[58]
	Water extraction	C. elegans	Antioxidant	ROS, MDA, NEFA, GSSG↓, SOD, CAT, GSH-Px, GSH↑	[57]
	Water extraction	Kunming mice	Anti-inflammatory	NO, iNOS, COX-2, TNF-*α* and IL-1*β*↓, SCFAs↑, TLR4 protein expression↓, ERK, JNK p38↓	[7]
Triterpenoid	Ethanol extraction	C57BL/6J mice, HepG2 cells	Ameliorate of insulin resistance	Upregulation of PI3K/Akt/GSK3*β*pathway	[34]
	Ethanol extraction	db/db mice, HepG2 and LO2 cells	Anti-inflammatory	IL-6, IL-1*β*, TNF-*α*↓, hepatic expression of Rho-kinase and NF-*κ*B↓	[51]
	Ethanol extraction	Mouse C2C12 myoblasts and 3T3-L1 preadipocytes	Improve glucose uptake	Activate AMPK-p38 pathway, Insulin sensitivity of adipocytes↑	[41]
	Ethanol extraction	Male Sprague-Dawley rats	Attenuates kidney injury	AMPK phosphorylation↑, mTOR phosphorylation↓	[96]
	Ethanol extraction	Male Sprague-Dawley rats	Hypolipidemic	Inhibition of intestinal apoB48 production through TNF-*α*/p38 MAPK signaling pathway, MDA↓, GSH-P, SOD, CAT↑	[85]
	Ethanol extraction	3T3-L1 adipocytes	Promote glucose uptake in 3T3-L1 adipocytes	AMPK activation↑, IKK*β* phosphorylation in adipocytes↓, restored insulin-mediated phosphorylation of IRS-1 tyrosine and Akt	[42]
	Ethanol extraction	FFA-induced HepG2 steatosis cells	Antioxidant	SOD↑, MAD↓	[59]
	Ethanol extraction	RAW 264.7 cells	Anti-inflammatory	NO, TNF, PGE2, IL-6↓, COX-2, iNOS, NF/p65↓	[48]
	Ethanol extraction	RAW264.7 cell	*α*-Glucosidase inhibitory and anti-inflammatory	mRNA expression of iNOS, COX-2, NF-*κ*B, IL-6, IL-1*β*, and TNF-*α*↓, protein expression of iNOS, NF-*κ*B/p65 and COX-2↓	[49]
Flavonoid	Hot water extraction	Male C57BL/6 J mice	Regulation of gut microbiota	Faecal microbiota diversity↑, Bacteroidetes↑, Firmicutes, Proteobacteria↓	[65]
	Ethanol extraction	RAW264.7 cell	Inhibit XOD activity, inhibition of NO production in LPS induced RAW264.7 cells	Not mentioned	[32]
Extract	Ethanol/chloroform extraction	KM male mice	Hypolipidemic	CYP7A1↑, HMG-CoA reductase↓	[83]
	Water extraction	Male Wistar albino rats	Alleviates diabetic nephropathy	Reduce oxidative stress, suppress the activation of the polyol pathway through aldose reductase inhibition	[14]
	Ethanol extraction	Kunming mice	Hypolipidemic	TNF-*α*, mRNA↓, p38 phosphorylation↓, inhibit MAPK signaling along the TNF-*α*/p38MAPK pathways	[84]
	Ethanol/water extraction	SD male rats	Antioxidant	MDA↓, SOD, GSH-Px↑	[93]
	Hot water extraction	Male C57/BL6J mice	Inhibit *β* cell apoptosis	Caspase-8, caspase-9, cleaved caspase-3↓, Bax/Bcl-2↓, p38, ERK and JNK phosphorylation↓, Akt phosphorylation↑	[74]
	Ethanol extraction	HepG2 cells	Attenuates hepatic lipid deposition	p-mTOR↓, Beclin↑, p-p70S6K↓, p62↓	[40]
	Water extraction	SHR/cp rats	Improving insulin signaling in the hypothalamus	m-PI3Kp85↑, p-Akt↑, p-FoxO1↑, POMC↑, NPC↓	[11]
	Ethanol extraction	db/db mice	Protect against diabetic cardiomyopathy	TNF-*α*, IL-1*β*, IL-6↓, Bcl-2↑, cle-caspase-3, cle-caspase-9, Bax↓	[100]
	Hot water extraction	Male Sprague-Dawley rats	Ameliorate diabetes	SOD↑, MDA↓, Ins1, Ins2↑, *β*-cell mass↑, Ddit4, Fgf21↑, DNA replication↑, cytochrome P450↓	[52]

## Data Availability

All data used and/or analyzed during the present study are available from the corresponding author on reasonable request.
